# Effects of Feed Mixed with Lactic Acid Bacteria and Carbon, Nitrogen, Phosphorus Supplied to the Water on the Growth and Survival Rate of White Leg Shrimp (*Penaeus vannamei*) Infected with Acute Hepatopancreatic Necrosis Disease Caused by *Vibrio parahaemolyticus*

**DOI:** 10.3390/biology10040280

**Published:** 2021-03-30

**Authors:** Linh Nguyen Thi Truc, Tuu Nguyen Thanh, To Tran Thi Hong, Day Pham Van, Minh Vo Thi Tuyet, Nghia Nguyen Trong, Minh Phan Cong, Diep Cao Ngoc, Phu Truong Quoc

**Affiliations:** 1 Tra Vinh University, 126, Nguyen Thien Thanh, Tra Vinh 87000, Vietnam; truclinh@tvu.edu.vn (L.N.T.T.); nttuu@tvu.edu.vn (T.N.T.); tthongto@tvu.edu.vn (T.T.T.H.); phvday@tvu.edu.vn (D.P.V.); tuyetminhcntc@tvu.edu.vn (M.V.T.T.); 2 Aquaculture Pharmacy Company Limited, 149/41, Hoang Van Thu Street, An Cu Ward, Ninh Kieu District, Can Tho 94000, Vietnam; nguyentrongnghiaapc@gmail.com (N.N.T.); infoapc.vn@gmail.com (M.P.C.); 3 Department of Aquatic Pathology, College of Aquaculture and Fisheries, Can Tho University, Ninh Kieu District, Can Tho 94000, Vietnam; cndiep@ctu.edu.vn

**Keywords:** acute hepatopancreatic necrosis disease (AHPND), CNP, LAB, white leg shrimp (*Penaeus vannamei*)

## Abstract

**Simple Summary:**

This study aimed to evaluate the growth, survival rate, and resistance to Acute hepatopancreatic Necrosis Disease (AHPND) of white leg shrimp (*Penaeus vannamei*) by using *Lactobacillus plantarum*, *Lactobacillus fermentum*, and *Pediococcus pentosaceus* mixed with feed, and at the same time supplying CNP in a ratio of 15:1:0.1 to the water. The result showed that shrimps were fed with feed containing lactic acid bacteria (LAB), especially L. plantarum have an effective to increased shrimp growth, stimulated non-specific immune system such as total hemocyte cells, granulocyte cells, hyaline cells, and protected shrimp to ANPND. The supply of CNP to the water have increased the intensity of *V. parahaemolyticus* effects on shrimp health; significantly decreased non-specific immune parameters of shrimp by 30–50%, therefore increased the AHPND infected rate and mortality of shrimp compared with without CNP group. In summary, LAB has a good effect to shrimp and the supply of CNP had significantly reduced the shrimp’s immune response and increased the susceptibility of shrimp to AHPND in both cases of use with and without LAB-containing diets.

**Abstract:**

This study aimed to evaluate the growth, survival rate, and resistance to acute hepatopancreatic necrosis disease (AHPND) of white leg shrimp (*Penaeus vannamei*) by using *Lactobacillus plantarum*, *Lactobacillus fermentum*, and *Pediococcus pentosaceus* mixed with feed, and at the same time supplying CNP in a ratio of 15:1:0.1 to the water. As a result, the treatments that shrimp were fed with feed containing lactic acid bacteria (LAB), especially *L. plantarum*, have increased shrimp growth, total hemocyte cells, granulocyte cells, and hyaline cells significantly (*p* < 0.05) in comparison to the control group. The supply of CNP to the water has promoted the intensity of *V. parahaemolyticus* effects on shrimp health and significantly decreased total hemocyte cells, granulocyte cells, and hyaline cells by 30–50% in the period after three days of the challenge, except in *L. plantarum* treatment, which had only a 20% decrease compared to other treatments. In CNP supplying treatments, the AHPND infected rate and mortality of shrimp were higher than those in other treatments. In summary, the supply of CNP had significantly reduced the shrimp’s immune response and promoted the susceptibility of shrimp to AHPND in both cases of use with and without LAB-containing diets.

## 1. Introduction

Acute Hepatopancreatic Necrosis Disease (AHPND) caused by *Vibrio parahaemolyticus* bacteria [1] has cost brackish shrimp farming over $1 billion a year [2] (Zorriehzahra, 2015). It appeared and caused mass deaths in farmed shrimp in the provinces of Soc Trang, Bac Lieu, Tra Vinh, and Ca Mau, Vietnam. In 2012, the total damaged farming area in the Vietnamese Mekong Delta was 39,000 ha [3]. AHPND continues to be a problem and has caused more severe damage and high mortality in some countries around the world [4]. In fact, Vietnamese farmers often use some products containing trehalose, glutamic acid, phosphate salts (supplying carbon (C), nitrogen (N), and phosphorus (*p*)), and some other amino acids as fertilizers for favoring the growth of phytoplankton and beneficial microorganisms. However, AHPND often occurs after 14 days of administering products containing trehalose, glutamic acid, and phosphate salts. The study of [5] showed that adding trehalose, glutamic acid, KH_2_ PO_4_, and K_2_ HPO_4_ to the experimental environment promoted the development of *V. parahaemolyticus*. Thus, it is hypothesized that the addition of trehalose, glutamic, and phosphate salts to the water may stimulate the growth of *V. parahaemolyticus* bacteria.

In order to restrain AHPND, lactic acid bacteria (LAB) mixed with feed counter *V. parahaemolyticus* bacteria are used, which is one of the methods used to improve shrimp growth, enhance the immune system, and prevent disease [6]. Lactic acid bacteria are widely used in human and animal probiotics to stimulate digestion and prevent a number of bacterial diseases. LAB have been selected as a supplement to aquatic animal feed because of many benefits, such as competing to eliminate pathogenic bacteria [4], supplying nutrients and digestive enzymes [7], and other benefits such as strengthening the immune system against pathogenic bacteria [4] and antiviral activities [8,9,10]. In the present study, LAB strains significantly reduced the mortality of shrimp challenged with *V. parahaemolyticus* from 70% in the positive controls to 11%, 22%, and 28% in the treatments administered the LAB of *Lactobacillus plantarum, Lactobacillus fermentum*, and *Pediococcus pentosaceus*, respectively.

In the present study, the target heterotrophic bacteria *V. parahaemolyticus* is the emergency pathogen on shrimp, and the carbon source used, trehalose, is the suitability nutrient for *Vibrio parahaemolytius* growth [5]. Vietnamese farmers often use commercial products containing trehalose to fertilize in the pond during pond preparation as fertilizers. AHPND frequently occurs after two weeks of using commercial products. The research aims to confirm the hypothesis of adding trehalose to water whether there is an increase in *Vibrio parahaemolyticus* density or not. In contrast, the target heterotrophic bacteria are beneficial microorganisms (e.g., *Bacillus*) that can improve shrimp growth performance and immune response. In addition, the main carbon source used by bacteria is molasses with the ingredient sucrose, which *V. parahaemolyticus* do not consume [11,12,13].

Based on the above, this experiment was conducted to test the hypothesis of whether the addition of carbon, nitrogen, and phosphorus (CNP) to a water environment stimulates the growth of *V. parahaemolyticus* bacteria or not, and if the three LAB strains selected to be added to the feed can improve growth, survival rate, composition, and the number of hemocyte cells, and also prevent AHPND in white leg shrimp (*P. vannamei*).

## 2. Materials and Methods

### 2.1. Water Source

Water used for the experiment was seawater collected from Ba Dong beach (Tra Vinh province, Vietnam), with a salinity of 28 ppt. Then, seawater was filtrated to remove sediments and disinfected with calcium hypochlorite at a concentration of 30 mg/L, vigorously and continuously aerated (within 24 h), examined, and eliminated of free chlorine content using Na_2_S_2_O_3_ at a ratio of 7:1 (Na_2_S_2_O_3_:Cl). After treating the seawater, it was diluted with tap water to obtain a 15 ppt brackish water.

### 2.2. Experiment Tank

The experimental tank systems were used, including twelve plastic tanks of one cubic meter tank (experiment 1) and twenty-four plastic buckets of 125 L (experiment 2). Composite tanks and plastic buckets were washed with clean water, then disinfected with 30 mg/L calcium hypochlorite and sun-dried for about 5 h before use.

### 2.3. Source of Experimental Shrimp

Post-larvae, 15 days old (PL15), bought from CP company, were tested by polymerase chain reaction (PCR) following the method of OIE (2009) and procedures of Sirikharin et al. (2014), with a specific primer for negative of white spot syndrome virus (WSSV), microsporidian disease, and AHPND. PL15 was nursed in the 2 cubic meter tanks at the wet lab of the School of Agriculture and Aquaculture, Tra Vinh University, until shrimp reached 3 g in size. Before setting up the experiment, the shrimp were examined again to select batches of shrimp negative of WSSV, microsporidia, and AHPND pathogens. After placing shrimp in experimental tanks, they were nursed for three days to adapt to environmental conditions before the experiment commenced.

### 2.4. Source of V. parahaemolyticus Bacterium

*V. parahaemolyticus* strain bears a virulent gene causing AHPND, which was isolated and stored in the lab of Tra Vinh University. This bacterium strain was recovered in nutrient broth medium (NB, Darmstadt, Merck), supplemented with 1.5% NaCl (NB^+^), then incubated at 28 °C for 18 h. The color and shape of the colony were recorded and the bacteria purity checked by gram stain. Pure bacteria colonies were cultured in NB^+^ medium at 28 °C for 18–24 h, and then bacteria density was measured using spectrophotometry with wavelengths at 610 nm.

### 2.5. Recovery, Culture of LAB, and Feed Preparation

*L. plantarum*, *L. fermentum*, and *p. pentosaceus* strains [8] that have the ability to inhibit the AHPND [8] were cultured in 100 mL of MRS broth medium (Darmstadt, Merck) supplemented with 1.5% NaCl for 48 h. Shortly after this, bacteria density was determined by OD measurement with an optical density of 1 ± 0.025, centrifuged at a rate of 7000 revolutions within 5 min, and rinsed thrice with sterile saline, and each bacteria strain was properly mixed into the commercial feed (CP brand name) containing 40% crude protein to reach a density of 10^8^ CFU/g, then coated with squid oil. Feed was put in labeled plastic bags and stored at a maximum temperature of 4 °C to be used for seven days. LAB density was reevaluated by the following procedure: about one gram of feed was weighed and put into a tube, and 9 mL of sterile saline was added, then ground and mixed. That solution was continuously diluted into various concentrations. One hundred microliters of these diluted solutions were spread on the MRS agar medium plate supplemented with 1.5% NaCl and then incubated at 28 °C for 48 h. LAB density (CFU/g) was determined by the formula below:

(1) $$\mathrm{Bacteria}\;\mathrm{density}\;\left(\mathrm{CFU}/g\right)=\;\frac{\frac{The\;number\;of\;colony\;\times \;dilution\;rate}{V}}{M}$$

where *V* is the volume of diluted solutions dripped onto MRS agar medium and *M* is the feed sample weight.

### 2.6. The Sources of CNP Supplied for the Experiment

Trehalose served as a C source. Glutamic acid (C_5_ H_9_ NO_4_) served as C and N source. KH_2_ PO_4_ and K_2_ HPO_4_ served as a source of *p*. The ratio of C:N:*p* used for the experiment was 15:1:0.1. The concentration of C, N, and *p* supplied to water was 3, 0.2, and 0.02 mg/L.

## 3. Research Methodology

### 3.1. The First Experiment

The first experiment was carried out to evaluate the effects of *L. plantarum, L. fermentum*, and *p. pentosaceus* on growth, survival rate, composition, and the number of hemocytes in white leg shrimp (*P. vannamei*).

#### 3.1.1. Experiment Set-Up

The experiment was a completely randomized design including four treatments and with three replicates for each. In the control treatment, shrimp were fed a normal diet, without containing LAB; for LAB1, LAB2, and LAB3 treatments, shrimp were fed with feed containing *L. plantarum, L. fermentum*, and *Pediococcus pentosaceus* at a density of 10^8^ Colony Forming Units per gam (CFU/g), respectively [8]. The experiment was arranged in 1000 L plastic tanks with a density of 100 shrimp/tank and a shrimp size of 3 g/shrimp. According to the guideline of the shrimp feed supplier, 3-g-weight shrimp should be fed 6% of body weight; therefore, in the present study, the feed was adjusted to 7% to meet shrimp demand and fullness. The tank water was properly aerated. Experimental shrimp were fed ad libitum (that is, 7% of shrimp weight) with feed containing 40% crude protein, four times a day at 7:00 and 11:00 a.m., and 3:00 and 9:00 p.m. After 1 h of feeding, uneaten feed was monitored by siphoning. The experiment was carried out for a period of 28 days due to a significant difference in the enhanced immunity system of shrimp-fed LAB on day 28.

#### 3.1.2. Observation and Sampling

Environmental factors were measured and adjusted to desirable ranges of pH (7.5–8.5), temperature (28–32 °C), NH_3_-N (less than 0.13 mg/L), and total alkalinity (120–180 mg CaCO_3_/L). The shrimp growth rate was determined by randomly capturing 10 shrimp and measuring their weight and length at a frequency every seven days. The hemolymph samples were collected three times to determine total hemocyte count (THC), granulocyte count (GC), and hyaline hemocyte count (HC). The hemolymph samples were collected at the beginning of the experiment; the second sampling was on the 15th day of the experiment, and the third sampling was at the end of the experiment. Hemolymph samples (100 µL) were collected from the ventral sinus and mixed gently with 900 µL of sterile anticoagulant (338 mM NaCl, 115 mM glucose, 30 mM trisodium citrate, 10 mM EDTA) solution. Thereafter, the number of total hemocyte cells was counted and calculated as cells/mL using a hemocytometer with a light microscope at 400× magnification [14]. For differential hemocyte, mixed hemolymph was centrifuged at 5000 rpm for 5 min at 4 °C, washed, and re-suspended with 200 µL of formalin-AS pH 4.6 (1:10). After centrifuge, the suspension (50 μL) was spread onto a glass slide and fixed by ethanol for 5 min, stained with Giemsa for 30 min, and washed in acetone and xylene [15]. Thereafter, the numbers of differential hemocyte cells were counted under a microscope.

### 3.2. The Second Experiment

The second experiment was conducted for determining the effects of LAB-containing diets and CNP supplied to the water on the survival rate and differential hemocyte and total hemocyte counts of white leg shrimp (*P. vannamei*) under the challenge with *V. parahaemolyticus* bacteria condition.

#### 3.2.1. Experiment Set-Up

The experiment was a completely randomized design with 10 treatments, each repeated thrice (30 experimental units). The experiment was conducted in a plastic bucket containing 100 L of 15 ppt brackish water under continuous aeration conditions. The shrimp of about 10 g/individual derived from the first experiment were used for the second experiment. For negative control (NC), negative control with CNP (NC + CNP), positive control (PC), and positive control with CNP (PC + CNP) treatments, twenty shrimp derived from the control treatment of the first experiment were stocked for each bucket. Similarly, the shrimp derived from LAB1 treatment of the first experiment were used for the LAB1 and LAB1 + CNP treatments of the second experiment; shrimp derived from the LAB2 treatment of the first experiment were used for the LAB2 and LAB2 + CNP treatments of the second experiment; and shrimp derived from the LAB3 treatment of the first experiment were used for the LAB3 and LAB3 + CNP treatments of the second experiment. Shrimp were fed ad libitum thrice daily at 7:00 a.m., 1:00 p.m., and 5:00 p.m. Shrimp were challenged with *V. parahaemolyticus*. Trehalose, glutamic acid, K_2_ HPO_4_, and KH_2_ PO_4_ were supplied to the water at a frequency of every seven days for a period of 14 days. After three days of the challenge, the water in the experimental tanks was changed 30% daily. The arrangement of the second experiment is described in Table 1.

#### 3.2.2. Observation and Sampling

Environmental factors were measured and adjusted following suitable range, pH (7.5–8.5), temperature (28–32 °C), NH_3_-N (less than 0.13 mg/L), and total alkalinity (120–180 mg CaCO_3_/L). pH and temperature were measured by Xylem Analytics Germany GmbH, and NH_3_-N and total alkalinity were determined by using phenate and titrimetric method, respectively. The density of *Vibrio* bacteria in shrimp gut lumen was determined four times during the experiment as follows before being challenged and at three, eight, and 13 days after being challenged with *V. parahaemolyticus*. The number of shrimp samples collected was three shrimp per tank. The hemolymph samples were collected twice during the experiment as follows before the challenge and on the third day of the challenge. Total hemocyte cells, granulocyte cells, and hyaline cells were counted. The number of total hemocyte cells and differential cells was counted and calculated as cell/mL with the same methods presented in the first experiment. The hepatopancreas samples of shrimp were collected in a period before being challenged with *V. parahaemolyticus*, and when shrimp showed signs of lethargy, stopped eating, and empty gut after three days of the challenge for histopathological analysis. Additionally, samples were collected (three shrimp/tank) at the end of the experiment.

#### 3.2.3. Bacteria Challenged

The challenge method was performed as described by [1]. The PirA gene of bacteria was examined by the AP3 method. Shrimps were immersed into *V. parahaemolyticus* solution at a density of 2 × 10^7^ CFU/mL for 15 min, then picked up and placed in the experimental tank containing *V. parahaemolyticus* bacteria at a density of 10^6^ CFU/mL. For negative control, shrimp were immersed in sterilized Tryptic soy broth medium supplemented with 1.5% NaCl (TSB, Merk, Darmstadt, Germany) for 15 min, then both shrimp and soaked solution were placed in the experimental tanks.

#### 3.2.4. Histopathological Analysis

Samples of hepatopancreas (HP) were extracted from the shrimp and fixed in Davidson’s AFA with a ratio of 1 hepatopancreas:10 Davidson’s AFA in 48 h (volume/volume). The HP samples were then placed in 70% alcohol as described by [16] before they were dehydrated by 70%, 80%, 95%, and 100% ethyl alcohol, and xylene. The HP samples were then cast with paraffin and sectioned into pieces with 5 μm thickness. The sectioned samples were put in 45–50 °C water before mounting on glass slides to make microscopic samples. The slides were stained with Haematoxylin và Eosin (H&E) before observation under a microscope.

### 3.3. Measurement of Growth Performance

The growth performance of shrimp was calculated using the formula given as:

Daily weight gain (g/day): DWG = (W_final_ − W_initial_)/t

Percent weight gain: PWG (%) = (W_final_ − W_initial_)/W_initial_ * 100

Daily length gain: DLG (mm/day) = (L_final_ − L_initial_/t

Percent length gain: PLG (%) = L_final_ − L_initial_/L_initial_ * 100

Where,

W_final_ and L_final_: mean final weight (g) and mean final length (mm) of shrimp at the end of the experiment;

W_initial_ and L_initial_: mean final length (g) and mean initial length (mm) of shrimp at the beginning of the experiment.

t: experimental period (day).

DLG was examined from tip of rostrum to tip of tail.

### 3.4. Survival Rate

Survival rate was determined following formula:

(2) $$\mathrm{Survival}\;\mathrm{rate}\;\left(\%\right)\;=\frac{\mathrm{number}\;\mathrm{of}\;\mathrm{surviving}\;\mathrm{shrimp}\;}{\mathrm{Total}\;\mathrm{shrimp}\;\mathrm{in}\;\tan k}\;\times \;100$$

### 3.5. Data Analysis

One-way ANOVA was applied to analyze the data of the experiments, in which Duncan’s Test was used to determine a significant difference of means between the treatments (*p* < 0.05). The data were presented by mean ± SE.

## 4. Results and Discussion

### 4.1. Results of the First Experiment

#### 4.1.1. The Effects of LAB-Containing Diets on the Growth of White Leg Shrimp (*P. vannamei*)

**Figure 1:**
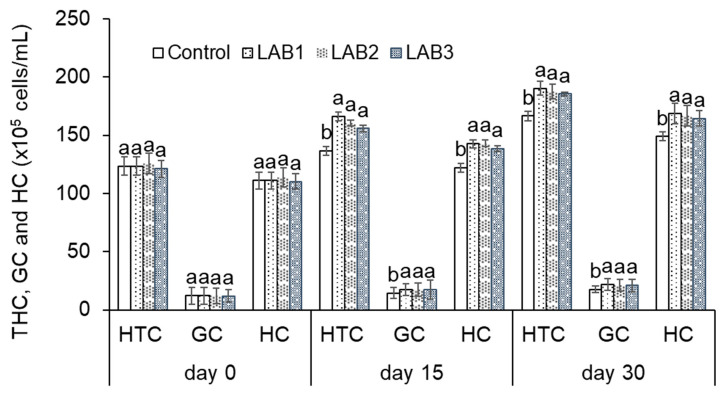
Total results of hemocyte cells counted in white leg shrimp (*Penaeus vannamei*): THC: total hemocytes count; GC: granulocytes count; HC: hyaline hemocytes count. Treatments with different letters were significantly different (*p* < 0.05). Error bar indicated standard error (SE) with three replications.

The experimental results showed that the growth of shrimp in length and weight was significantly different between the control treatment and other treatments (LAB1, LAB2, and LAB3), while there was no significant difference in growth between LAB1, LAB2, and LAB3 treatments. The mean weight of shrimp between treatments was not significantly different on the 21st day of the experiment. However, at the fifth sampling (the 28th day), it was significantly different between the treatments. In the treatment LAB1, shrimp showed the highest mean weight (12.7 ± 0.29 g), followed by treatment LAB3 (12.3 ± 0.31), and these were significantly different (*p* < 0.05) compared to the control treatment (11.27 ± 0.39 g). Similarly, the mean length of shrimp was also highest in LAB1 treatment (11.9 ± 0.29 mm), followed by LAB3 (11.7 ± 0.39 mm), after the 28-day experiment (Figure 1). The growth of shrimp fed with LAB-containing diets has been improved with an increase of about 3–11% of DWG and 3.7–8.9% of DLG. The PWG of shrimp in LAB1, LAB2, and LAB3 treatments has increased by 83.4%, 64.2%, and 73%. The PLG of shrimp has increased by 26%, 17%, and 20.84% in comparison to those in the control treatment. Especially, the diet containing *L. plantarum* (LAB1) led to the best WG, LG, DWG, and DLG after 28 days of the experiment.

#### 4.1.2. The Effects of of LAB-Containing Diets on Survival Rate of White Leg Shrimp (*P. vannamei*)

After 30 days of the experiment, the survival rate of shrimp reached over 90% in all treatments, and the difference was not statistically significant among them. However, treatment with the highest survival rate was LAB1 (99.4%), while the lowest was in the control treatment (97.2%).

#### 4.1.3. The Effects of LAB-Containing Diets on Differential Hemocyte Count and Total Hemocyte Count of White Leg Shrimp (*P*. *vannamei*)

Immune response of shrimp was determined by total number of hemocyte cells, granulocyte cells, and hyaline cells. The experimental results were shown in Figure 1.

In general, the results showed an increase of THC, GC, and HC of shrimp during the time of the experiment. The experimental results indicated that THC, GC, and HC of shrimp in the LAB1, LAB2, and LAB3 treatments were quite high and significantly different in comparison to those in the control treatment. After 15 days of the experiment, the highest THC, GC, and HC were 166.1, 17.4, and 142.8 × 10^5^ cells/mL, respectively, in the LAB1 treatment. Similarly, THC, GC, and HC of shrimp continuously increased and reached 190.3, 21.8, and 168.6 × 10^5^ cells/mL, respectively, in LAB1 treatment after 30 days of the experiment. Meanwhile, THC, GC, and HC of shrimp in NC treatment were 136.3, 14.5, and 121.9 × 10^5^ cells/mL after 15 days of the experiment, and 166.5, 17.7, and 148.9 × 10^5^ cells/mL after 30 days of the experiment. It could be concluded that using *L. plantarum*, *L. fermentum*, and *p. pentosaceus* has the potential to enhance the innate immunity of white leg shrimp (*P. vannamei*), especially *L. plantarum*.

### 4.2. Results of the Second Experiment

#### 4.2.1. The Effects of the LAB-Containing Diets and CNP Supplied to the Water on Differential Hemocyte Count and Total Hemocyte Count of White Leg Shrimp

Experimental results showed that THC, GC, and HC of shrimp at the beginning of the experiment were significantly different between the control treatments and the treatments fed shrimp with LAB-containing diets. Because shrimp derived from the first experiment were used for the second experiment, the treatments in which shrimp were fed with LAB-containing diets have stimulated shrimp immunity; thus, THC, GC, and HC of shrimp were higher than those of the treatments that shrimp were fed with normal feed.

**Figure 2:**
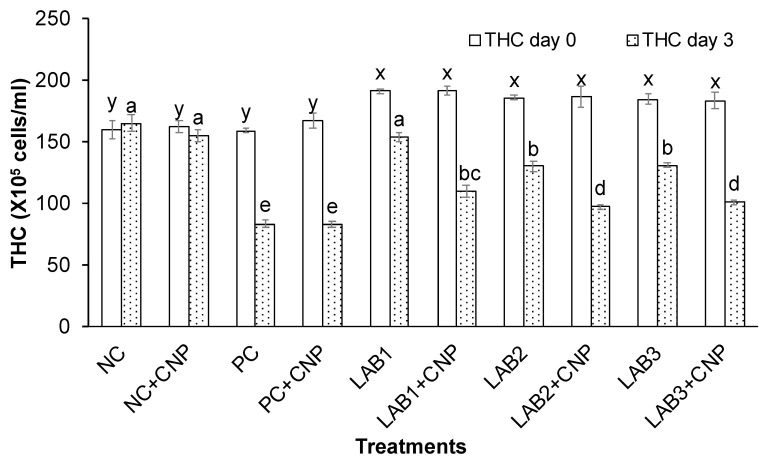
Total hemocytes count. The treatments with different letters (x, y or a, b …) were significantly different (*p* < 0.05). Error bar indicated standard error (SE) with three replications.

**Figure 3:**
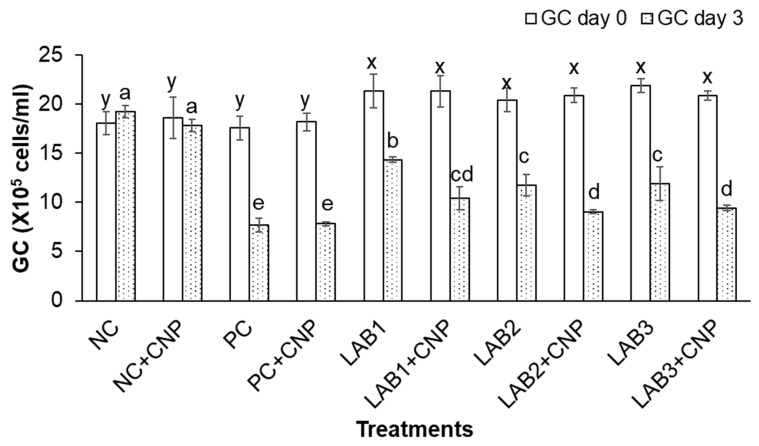
Granulocytes count. The treatments with different letters (x, y or a, b …) were significantly different (*p* < 0.05). Error bar indicated standard error (SE) with three replications.

**Figure 4:**
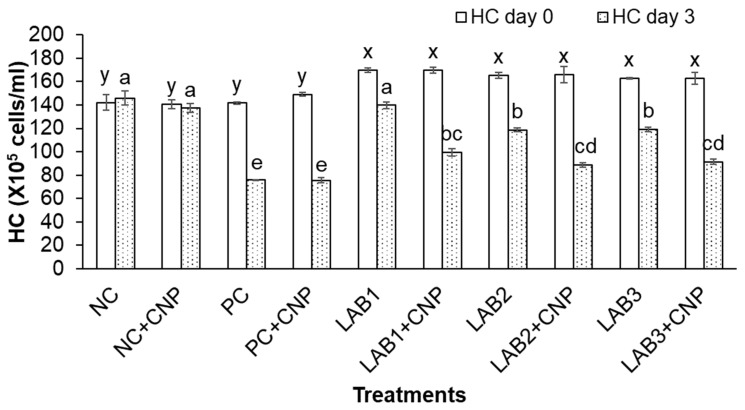
Hyaline count. The treatments with different letters (x, y or a, b …) were significantly different (*p* < 0.05). Error bar indicated standard error (SE) with three replications. NC: negative control, NC + CNP: negative control with CNP, PC: positive control, and PC + CNP: positive control with CNP.

After three days of the challenge with *V. parahaemolyticus* bacteria, the THC, GC, and HC of challenged shrimp in all treatments were significantly reduced in comparison to NC treatment. Especially, THC, GC, and HC of shrimp in PC, PC + CNP treatments were decreased about 50% in comparison to those at the beginning of the experiment. Meanwhile, THC, GC, and HC of shrimp in NC treatment were increased 2.7–6.5%, and THC, GC, and HC of shrimp in NC + CNP were decreased 2.5–4.4% (Figure 2, Figure 3 and Figure 4). Thus, the shrimp immunity could be declined under conditions of shrimp infected with *V. parahaemolyticus* bacteria.

Similar to the first experiment, THC, GC, and HC of shrimp in the treatments in which shrimp were fed with LAB-containing diets (LAB1, LAB2, and LAB3) were significantly higher than those of the treatments. Shrimp were fed with normal feed (PC and PC + CNP) under the conditions of shrimp challenged with *V. parahaemolyticus* bacteria. THC of shrimp in LAB1, LAB2, and LAB3 treatments was decreased by 19.4–29.2%; GC of shrimp in LAB1, LAB2, and LAB3 treatments was decreased by 32.8–45.5%; and HC of shrimp in LAB1, LAB2, and LAB3 treatments was decreased by 17.7–28.2%. Meanwhile, THC, GC, and HC of shrimp in PC and PC + CNP treatments were decreased by 47.6–50.2%, 56.2–57.1%, and 46.4–49.3%, respectively. Therefore, the immunity of shrimp was improved when shrimp were fed LAB-containing diets, in which *L. plantarum* bacteria have the best efficiency in improving shrimp immunity.

When comparing the treatments with and without CNP supplied to the water, under the condition of being challenged with *V. parahaemolyticus* bacteria, THC, GC, and HC of shrimp in the treatments with CNP supplied (LAB1 + CNP, LAB2 + CNP, and LAB3 + CNP) were lower than those of the treatments without CNP supplied (LAB1, LAB2, and LAB3), and the difference was significant. THC of shrimp in LAB1 + CNP, LAB2 + CNP, and LAB3 + CNP was decreased 42.5–47.7%; GC of shrimp in LAB1 + CNP, LAB2 + CNP, and LAB3 + CNP was decreased 51.1–56.7%; and HC of shrimp in LAB1 + CNP, LAB2 + CNP, and LAB3 + CNP was decreased 41.4–46.7%. Meanwhile, THC, GC, and HC of shrimp in LAB1, LAB2, and LAB3 treatments were decreased 19.4–29.2%, 32.8–45.5%, and 17.7–28.2%, respectively. The results showed that CNP could intensify the effects of *V. parahaemolyticus* on the hemocyte of shrimp.

#### 4.2.2. The Effects of LAB-Containing Diets and CNP Supplied on Survival Rate of Shrimp

##### Survival Rate of Shrimp

**Figure 5:**
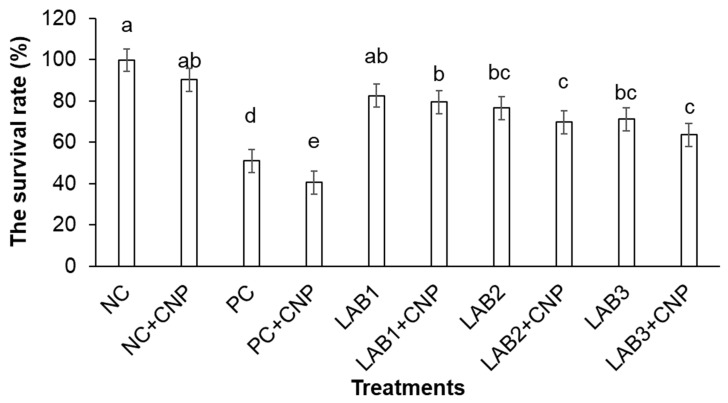
White leg shrimp (*Penaeus vannamei*) survival after 14 days of challenge. Note: Treatment with different letters was significantly different (*p* < 0.05). Error bar indicated standard error (SE) with three replications.

The study results (Figure 5) showed that the survival rate of shrimp in NC was very high (98.3%), which proved shrimp used in the experiment were completely healthy. Meanwhile, the survival rates of shrimp in all treatments challenged with *V. parahaemolyticus* bacteria were lower than those of the NC treatment, and the difference was statistically significant except for the LAB1 treatment. The survival rate of shrimp in PC treatment was only 40%; it also proved that *V. parahaemolyticus* has high virulence and causes mortality of shrimp in challenged treatments.

The survival rate of shrimp in treatments fed with LAB-containing diets varied from 63.3% to 83.3% and was higher than those of PC treatments. The difference in shrimp survival rate between treatments fed with LAB-containing diets and the PC treatment was statistically significant. LAB-containing diets were able to improve shrimp survival under conditions of shrimp challenged with *V. parahaemolyticus* bacteria.

In the water that was supplied with CNP, the survival rate of shrimp appeared to be reduced compared to the survival rate of shrimp in the treatments not supplied with CNP. Only the difference in shrimp survival rate in the pair of PC and PC + CNP treatments was statistically significant. Meanwhile, the difference in shrimp survival rate between the other pairs of treatments (LAB1 and LAB1 + CNP; LAB2 and LAB2 + CNP; LAB3 and LAB3 + CNP) was not statistically significant.

##### Histology of Shrimp Hepatopancreas

**Figure 6:**
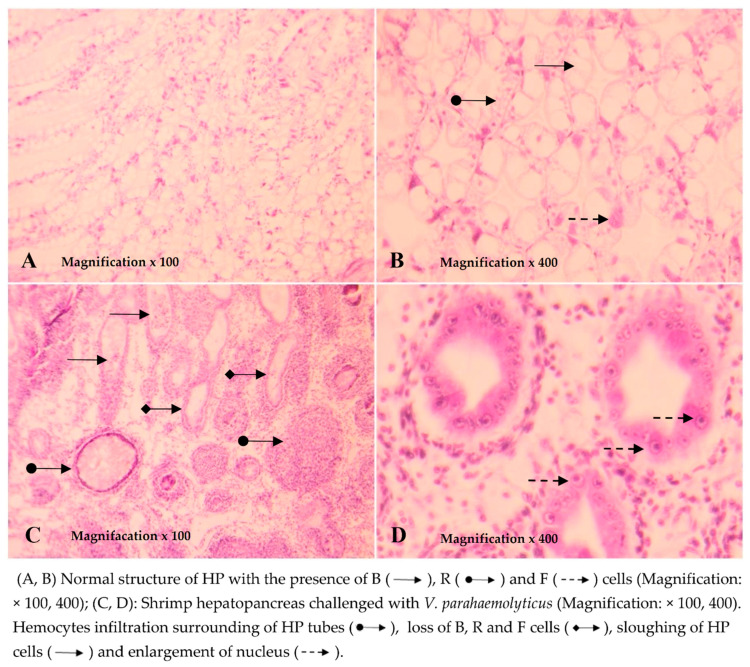
Histological structure of shrimp hepatopancreas.

The results of the histopathological analysis showed that the hepatopancreatic tissues of the shrimp before the challenge had a normal structure in the presence of B, R, and F cells (Figure 6A,B). Similarly, the structure of hepatopancreatic tissues of shrimp in the NC and NC + CNP treatments during the time of the experiment was also normal. Meanwhile, the hepatopancreatic tissue of shrimp three days after being challenged with *V. parahaemolyticus* bacteria showed an abnormal structure with typical histological symptoms of AHPND, which was similar to the description by Lightner et al. (2012). HP tissues showed a lack of number and dysfunction of B, R, and F cells, sloughing of HP tubule epithelial cells, prominent karyomegaly, massive hemocyte aggregation, and formation of melanized granulomas (Figure 6C,D).

Based on the histopathological analysis of 18 challenged shrimp in the first seven days, the incidence of AHPND in the treatment PC and PC + CNP were the highest at 77.8% and 88.8%, respectively. In treatments in which shrimp were fed with LAB-containing diets, the incidence of shrimp was lower than those of shrimp in positive control treatments. The incidence of AHPND of shrimp in LAB1, LAB1 + CNP, LAB2, LAB2 + CNP, LAB3, and LAB3 + CNP treatments after three days of the challenge were 22.2%, 33.3%, 33.3%, 55.6%, 44.4%, and 55.6% respectively.

Results of analysis of hepatopancreatic tissues of the shrimp that survived to the end of the experiment showed that there were 60% and 66.7% of shrimp in the positive control treatments (PC and PC + CNP) that showed signs of AHPND disease. Meanwhile, the incidence of shrimp in LAB1, LAB1 + CNP, LAB2, LAB2 + CNP, LAB3, and LAB3 + CNP treatments that showed signs of AHPND disease were 0%, 11.7%, 13.3%, 16.7%, 18.3%, and 20%, respectively.

In general, it is possible that the water that was supplied with CNP has intensified the effects of *V. parahaemolyticus* bacteria on shrimp. Shrimp that were fed LAB-containing diets were able to reduce the morbidity and mortality of shrimp under the condition of being challenged with *V. parahaemolyticus*, especially the *L. plantarum* containing diet.

### 4.3. Discussion

The results of this study showed that, with the diets containing *Lactobacillus plantarum*, *L. fermentum*, and *Pediococcus pentosaceus* bacteria at a dose of 10^8^ CFU/g, the growth of shrimp has been improved in comparison to a normal diet. The study has provided additional scientific information on the effectiveness of *L. plantarum*, *L. fermentum*, and *p. pentosaceus* and confirmed the best effect of *L. plantarum* on the growth of white leg shrimp. The above results were consistent with those of [17] in a study of four micro-bound diets that were formulated to contain fermentation supernatant (FS), live bacteria (LB), dead bacteria (DB), and cell-free extract (CE) of *L. plantarum*, and exhibited significant differences (*p* < 0.05) in weight gain rate (WGR), specific growth rate (SGR), and feed conversion ratio (FCR) compared to the control group. In the studies of [18,19], shrimp that were fed with heat-killed *Lactobacillus plantarum* strain L-137 (HKL-137) and LP20 product, which contains 20% heat-killed *L. plantarum* strain L-137 (HK L-137) and 80% dextrin in dried-weight basis heat-killed *L. plantarum* at dosage 0.5 g and 1 g/kg, showed higher weight, length, and specific growth rate (SGR) than those fed basal diet in both phase PL1–15 and PL15–45. In other research, white leg shrimp that were fed with Artemia immersed in 2.6 × 10^6^ CFU/mL of *L. plantarum* and *L. casei* bacteria solutions showed a significant difference in the growth and survival rate compared to the control, and growth and survival rate of shrimp between the *L. plantarum* and *L. casei* treatments were not significantly different [20].

Thus, products from LAB, especially *L. plantarum*, including live bacteria, dead bacteria, cell-free extracts, and enriched feed (*Artemia* immersed in bacterial solution), all have the effect on improving growth, survival rate, and FCR of shrimp. The dietary content of probiotics has been hypothesized to enhance the appetite or stimulate the digestibility of organisms. Additionally, it has revealed possible involvement of probiotics in the improvement of the intestinal microbiota balance as well as in the production of extracellular enzymes, which by turns enhance the feed utilization and growth of the cultured species, as they act as growth promoters [17,21,22].

Along with positive effects in stimulating weight gain, usage of *L. plantarum, L. fermentum*, and *p. pentosaceus* also has positive effects on stimulating the non-specific immune system of white leg shrimp. Interestingly, in the presence of *L. plantarum*, *L. fermentum*, and *p. pentosaceus*, blood cell count and white blood cell types of the shrimp increased. LAB-containing diets have caused an increase in THC, GC, and HC of shrimp, which was similar to the results recorded in some previous studies on white leg shrimp fed *L. plantarum* [23] and heat-killed *L. plantarum* strain L-137 [19], and on giant freshwater shrimp (*Macrobrachium rosenbergii*) fed *L. plantarum* MTCC1407 and heat-killed *L. plantarum* MTCC1407 [24,25,26]. Blood cells play an important role in shrimp’s non-specific immune system with a specific function for each type of cells, such as hyaline and granulocytes, with a participating role in the process of coagulation and phagocytosis. Hyaline hemocytes absorb pathogens or foreign particles through the process of phagocytosis, and they also intervene in the coagulation process. Granulocytes, through encapsulation, nodule formation, and cytotoxicity, destroy invading elements, and they also intervene in melanization (ProPO system) [27,28,29]. Hence, an increasing total hemocyte count, as well as hyaline and granulocyte, will contribute to the improvement of some cellular and humoral immune responses in shrimp against pathogenic bacteria.

Acute hepatopancreatic necrosis disease (AHPND) is a serious disease in shrimp caused by *V. parahaemolyticus* bacteria carrying virulent PirA and PirB genes. The application of biological methods is effective to limit drug resistance of pathogens, and usage in disease prevention is increasingly popular. Previous studies showed the effectiveness of using LAB to limit harmful effects of this disease, such as the study of [8] using *L. plantarum* T8 and T13 bacteria and the study of [18] using heat-killed *L. plantarum* strain L137. Similarly, *L. plantarum*, *L. fermentum*, and *P. pentosaceus* supplementation of the present study demonstrated a strong resistance against *V. parahaemolyticus*. Specifically, the survival rate of the shrimp at the supplemented treatments was 20–30% higher than that of the control in all challenging treatments. Hereby, *L. plantarum*, *L. fermentum*, and *p. pentosaceus* containing diets brought about high protection for shrimp cultured from AHPND with the best efficiency in treatments using *L. plantarum*. Similar results were also noted in a number of studies with other pathogens; for example, dietary inclusion of *L. plantarum* significantly increased disease resistance of Pacific white shrimp and giant freshwater prawn against *V. aginolyticus*, *V. harveyi*, and *A. hydrophila*, respectively [23,25,30,31]. In the case of *P. pentosaceus*, dietary inclusion significantly increased disease resistance of *L. vannamei* and *Haliotis discus* hannai against *V. vulnificus*, *V. rotiferianus*, *V. campbellii*, and *V. parahaemolyticus*, respectively [17,32,33], and the administration of *L. acidophilus* and *Lactobacillus* sp. significantly enhanced disease resistance of *L. vannamei* against *V. alginolyticus* and WSSV, respectively [34,35]. It is well-acknowledged that anti-pathogenic effect of LAB is due to the antagonistic activity of lactic acid bacteria used against harmful strains of bacteria through competitive mechanisms [32]. Probiotics produce a wide range of bioactive molecules, which possess bactericidal activity such as antibiotics, bacteriocins, siderophores, enzymes (lysozymes, proteases), and/or hydrogen peroxide as well as organic acids. They inhibit pathogenic bacteria in the host’s intestinal tract and provide a barrier against the proliferation of pathogens [21,36,37].

AHPND in shrimp usually occurs very early in the crop, 10–35 days after stocking; during this period the nutrient content in water is usually very low. In fact, farmers often apply some fertilizers and probiotics, which contain some sources of carbon, nitrogen, and phosphorus such as trehalose, inorganic nitrogen, amino acids, and inorganic phosphates at the beginning of the crop. Trehalose sugars are synthesized from bread yeast (*Saccharomyces cerevisiae*) [38,39,40], and they are used as a filler in fertilizers for favoring phytoplankton or probiotics. Research by [5] showed that *V. parahaemolyticus* strain 12 can grow on media containing trehalose, L-glutamic, KH_2_ PO_4_, and K_2_ HPO_4_. This present study showed that the survival rates of shrimp in the treatments that supplied CNP were lower than the treatments that did not provide CNP; especially, the survival rate of shrimp between the two treatments PC and PC + CNP was significantly different. The survival rate of shrimp in the PC + CNP treatment decreased by about 11% compared with the survival rate of shrimp in the PC treatment (Figure 5). This indicates that the supply of CNP to the water increased the cell density of *V. parahaemolyticus*; hence, CNP treatments had indirect impact on white leg shrimp. It is clear that using CNP 15:1:0.1 supplied to water increases heterotrophic bacteria number, including *V. parahaemolyticus* [11,12]. Carbon, nitrogen, and phosphorus are important nutrient elements of living organisms with functions: a constituent of cellular material, amino acids, nucleic acids nucleotides, coenzymes nucleotides, phospholipids, lipopolyshacrarides, and teichoic acids, especially heterotrophic bacteria. The supply of CNP will stimulate the growth of heterotrophic bacteria and other microorganisms in pond/tank water, including *Vibrio* sp. [41]. According to [42], C and N application to the pond with the corresponding ratio of 15:1 increased total bacteria and *Vibrio* sp. In the present study, supplementation of C, N, and P (15:1:0,1) to the water environment provided nutrients for *V. parahaemolyticus* to grow quickly; consequently, *V. parahaemolyticus* grew about triple in the CNP treatments than in the treatments without CNP, and caused greater effects on cultured shrimp. In contrast, probiotic applications to CNP and *V. parahaemolyticus* treatments in the present study showed that all *L. plantarum, L. fermentum*, and *p. pentosaceus* alleviated the impacts of *V. parahaemolyticus* on shrimp. Especially, the supply of *L. plantarum* in feed had the best effect on AHPND resistance of shrimp compared to two other potential probiotics.

## 5. Conclusions and Recommendations

The LAB-containing diets, especially *L. plantarum* strain, have significantly improved immune response in white leg shrimp (*P. vannamei*). The supply of trehalose, glutamic acid, KH_2_ PO_4_, and K_2_ HPO_4_ at a ratio of C:N:*p* as 15:1:0.1 in the experimental water caused increasing shrimp mortality and likelihood of AHPND outbreak in white leg shrimp (*P. vannamei*). However, the addition of *L. plantarum* to feed significantly reduced the mortality, as well as AHPND infection rate in the presence of CNP. Hence, *L. plantarum* can be a candidate to reduce AHPND in intensive shrimp farming. Further studies should be conducted to determine the resistance to AHPND of *L. plantarum* strains in intensive white shrimp farming ponds.

## Figures and Tables

**Table 1:** Experimental description.

Treatments	LAB Strain Mixed with Feed (10^8^ CFU/mL)	CNP Supplied to the Water at a Ratio of 15:1:0.1	Challenge with *V. parahaemolyticus* (10^6^ CFU/mL)
NC	No	No	No
NC + CNP	No	Yes	No
PC	No	No	Yes
PC + CNP	No	Yes	Yes
LAB1	*L. plantarum*	No	Yes
LAB1 + CNP	*L. plantarum*	Yes	Yes
LAB2	*L. fermentum*	No	Yes
LAB2 + CNP	*L. fermentum*	Yes	Yes
LAB3	*p. pentosaceus*	No	Yes
LAB3 + CNP	*p. pentosaceus*	Yes	Yes

Notes: NC: negative control; LAB1: *Lactobacillus plantarum*; LAB2: *Lactobacillus fermentum*; LAB3: *Pediococcus pentosaceus*; CNP: carbon, nitrogen, and phosphorus supplied.

## Data Availability

Not applicable.
